# Scrambler therapy for the treatment of neuropathic pain related to leukemia in a pediatric patient: A case report

**DOI:** 10.1097/MD.0000000000008629

**Published:** 2017-11-10

**Authors:** Hahck Soo Park, Won-Joong Kim, Hyung Gon Kim, Seung Hee Yoo

**Affiliations:** Department of Anesthesiology and Pain Medicine, School of Medicine, Ewha Womans University, Ewha Womans University Mokdong Hospital, 1071 Anyangcheon-ro, Yangcheon-gu, Seoul, Republic of Korea.

**Keywords:** child, leukemia, neuropathic pain

## Abstract

**Rationale:**

Cancer-related neuropathic pain often responds poorly to standard pain treatments. Scrambler therapy has relieved refractory chronic pain in several uncontrolled clinical trials.

**Patient concerns:**

An 11-year-old female patient was suffering from left groin and medial thigh pain after irradiation to the knee. The girl was diagnosed with precursor B-cell lymphoblastic leukemia 2 years ago. Extramedullary relapse of leukemia developed 1 month ago and pain had started. She was treated with oral medications, but she was continuously complaining of severe pain.

**Diagnosis:**

Neuropathic pain caused by obturator nerve involvement in leukemia.

**Intervention:**

Scrambler therapy.

**Outcome:**

Pain reduction.

**Lessons:**

Scrambler therapy is noninvasive, is not associated with any complications, causes minimal discomfort during treatment, and is very effective in a pediatric patient with cancer-related neuropathic pain.

## 1 Introduction

Among the various types of cancer pain, cancer-related neuropathic pain occurs at a prevalence of at least 35% to 40% in patients.^[1]^ Also, cancer-related neuropathic pain often responds poorly to standard pain treatments, since the pain origin and mechanism are combined to produce a mixed mechanism pain, which may differ from classical neuropathic pain.^[2]^ Poor pain management is a common problem in children with cancer, although a growing body of research deals with pain and analgesia in children. Several reasons have been identified for the under treatment of pain in children with cancer, including the impression that children are less sensitive to pain than adults, or that infants do not experience pain,^[3]^ and the myth that the use of potent opioids will lead to a high risk of addiction in children.^[4,5]^

Scrambler therapy (ST) is an Food and Drug Administration-cleared treatment for neuropathic pain supported by multiple trials.^[6]^ Although the mechanism of ST is not yet clear, it may work by “scrambling” afferent pain signals and replacing them with synthetic “no-pain” information via the cutaneous nerves by the application of surface electrodes around the surface of painful areas in a noninvasive manner. ST has relieved refractory chronic pain in several uncontrolled clinical trials: in 11 cancer patients with abdominal pain^[7]^; in 226 patients with neuropathic pain including those with failed back surgery syndrome, brachial plexus neuropathy, and others^[8]^; refractory, chemotherapy-induced neuropathic pain^[9]^; a wide spectrum of cancer-related pain^[10]^; and postherpetic neuropathy,^[11]^ spinal cord stenosis, and failed back syndrome.^[12]^

We recently treated a pediatric patient who was suffering from neuropathic pain caused by obturator nerve involvement in leukemia and the patient experienced pain relief. To the best of our knowledge, this is the first reported case of a pediatric patient with neuropathic pain treated by ST.

## 2 Case presentation

An 11-year-old female patient with a height of 136 cm and a weight of 33 kg was referred from the pediatric unit for left groin and medial thigh pain with irradiation to the knee. The girl was diagnosed with precursor B-cell lymphoblastic leukemia 2 years ago and had received chemotherapy. Extramedullary relapse of leukemia developed 1 month ago and pain had started to cause interruption of normal activities. The pain was described as numbness, throbbing, and tightness. The pain was accentuated by palpation of adductor muscles. At the time of referral, her pain intensity was 8/10 [numerical rating scale (NRS) pain score].

**Figure 1:**
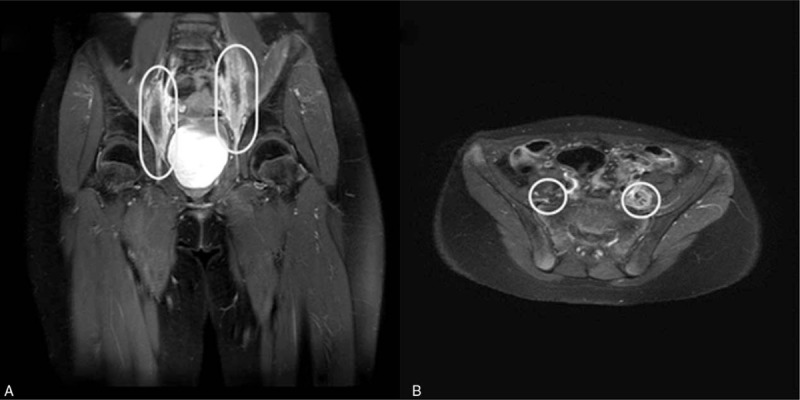
Magnetic resonance imaging scans. (A) Coronal image and (B) axial image.

The pelvic magnetic resonance imaging showed a tubular structure with increased diameter in the posterior aspect of the left psoas muscle at the L3–4 intervertebral disc level and the posterior aspect of the right psoas muscle at the L4–S1 intervertebral disc level. These lesions were thought to be connected to the part considered the obturator nerve. The enhancement was present toward the peripheral perineural sheath and around the perineural fat plane. Subtle enhancement was also seen in the adjacent psoas muscle. It was accompanied by perifascicular enhancement. These findings suggested a lesion involving the obturator nerve and the possibility of involvement due to acute leukemia in the extramedullary pattern (Fig. 1).

**Figure 2:**
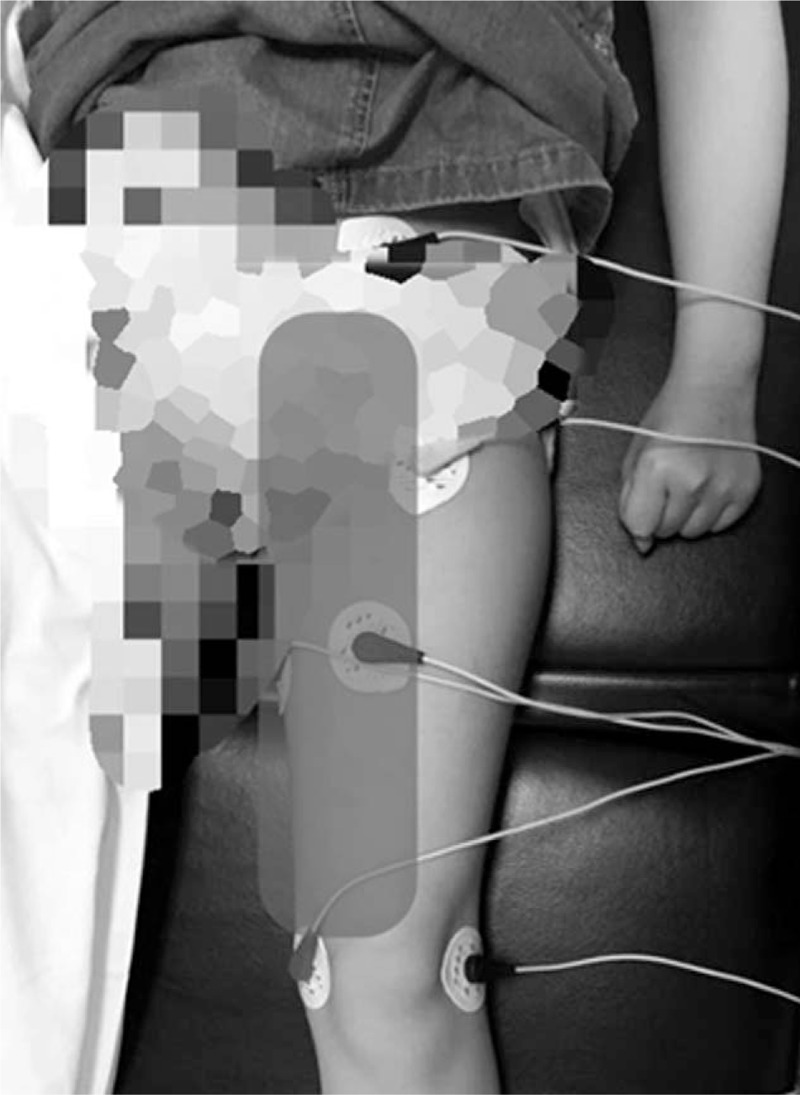
Patient's painful area and sites where the electrodes were attached.

Before visiting the pain clinic, the girl had been treated with oral gabapentin 300 mg/d, fentanyl patch 12 mcg/h, and naproxen (correct dosage for age); however, the girl was continuously complaining of severe pain. The pediatric unit requested us to perform the obturator nerve block, but both the patient and the caregiver were afraid of injections. Hence, ST was planned. Because the patient's pain was located in the groin area, medial thigh, and knee, the scrambler electrodes were attached to 3 normal sensory areas around the painful area for treatment (Fig. 2). We set up a 45-min daily treatment session for 4 consecutive days, at the same time and provided by the same physician.

During treatment, the NRS score decreased from 8/10 to 3/10 after the first session. Subsequent sessions were followed by marked improvement of pain: after 3 treatment sessions, NRS score was 0/10. Following pain reduction, drugs were progressively reduced and then prescribed at need. No other treatment sessions were performed. No adverse events were observed. We evaluated her pain control by phone. Pain intensity was investigated 1 and 4 weeks after completion of treatment: the patient referred to no pain.

## 3 Discussion

In the above case, ST was demonstrated to be an effective treatment in patients complaining of severe cancer-related neuropathic pain who were unable to experience relief from medication therapy. To the best of our knowledge, no data are available in the literature about the use of ST in treating neuropathic pain in children.

Cancer-related pain results from the disease and/or from the consequences of antineoplastic treatments. Neuropathic pain caused by the neuropathic mechanisms to produce a mixed mechanism pain,^[13]^ which may be different from the classical neuropathic pain, is more difficult to manage. Previous studies of ST including cancer patients only confirmed a lower reduction in the pain score compared with the studies including patients with noncancer pain.^[9,14]^ All studies on ST for managing cancer-related pain have been conducted in patients with chemotherapy-induced peripheral neuropathy,^[9,10,14]^ except for 1 case report which showed that ST was effective in managing metastatic bone pain in 2 patients.^[15]^ However, all studies on ST for managing cancer-related pain have been conducted in adult patients. Our case showed that ST is very effective in pediatric patients with cancer-related neuropathic pain.

The mechanism of ST is not clear, but Marineo et al^[16]^ suggested that electrical stimulus by electrodes gives “no-pain” information to peripheral receptors; C-fibers and Aδ fibers lead the stimulus to the central nervous system that receives it and reduces the pain symptoms. During ST, patients can refer no-pain sensations in the pain area, such as pressure and itching. The procedure for ST starts with a clear identification of the pain area. After this, electrodes are attached along the dermatome of the pain area, not on pain sites. After every treatment, before starting the next one, it is necessary to evaluate the pain areas again: the painful area can change and electrodes must be attached in a different way. After the placement of electrodes, electrical stimuli are applied. Intensity is gradually increased to the maximum value tolerated by the patient. This stimulus must not cause any additional pain or discomfort.^[16]^ We used ST according to the literature best practice.^[16]^ Pain alleviation should be maintained continuously for several days or several months after completion of treatment. The mechanism signifies that remodulation occurs in the peripheral and central nervous systems or the calcium channels of the synapses, which become the main target for treating neuropathic pain.^[16]^ Our patient also reported no pain after 1 and 4 weeks.

Other methods for treating chronic pain using an electrical stimulus are spinal cord stimulator (SCS) and transcutaneous electrical nerve stimulation (TENS). Many hypotheses have been put forward to explain the treatment mechanisms of SCS and TENS, such as supraspinal processes, modulation of the descending inhibitory pathways, peripheral release of calcitonin, increased gate control for pain threshold, reduction of the windup phenomenon, and reduction in impulses from damaged nerves.^[9,17,18]^ ST is very similar to existing TENS as it treats pain by applying an electrical stimulus from electrodes attached to the skin. However, ST shows differences from TENS in the method of applying an electrical stimulus and the treatment effect. First, TENS positions the electrodes on the area of pain, while ST positions the electrodes on normal sensory areas surrounding the area of pain. Second, TENS conveys unchanging on-off biphasic electric waves, whereas ST provides 16 nonlinear waveforms that change continuously.^[19]^

Peripheral nerve block (PNB) is now commonly incorporated into neuropathic pain management and the consequences of expanded use of PNB include improvement in pain relief and opioid requirements. Studies about major complications of PNB in pediatric patients have not been reported and adverse events were detected in 1% of cases who received lower extremity blocks.^[20]^ Despite these advances, it has introduced a new set of technical difficulties, patient education needs, and complications. Potential risks of PNB include vascular puncture and bleeding, nerve damage, and local anesthetic systemic toxicity.^[21,22]^ The limitation of single PNB is the short duration of action of most local anesthetics.^[23]^ In our case, the girl not only had fear of a nerve block, but long analgesic duration was also needed. Therefore, we thought that ST can be a good alternative method.

We report that a pediatric patient with cancer-related neuropathic pain was effectively treated by ST. ST is noninvasive, is not associated with any complications, causes minimal discomfort during treatment, and is similar or superior to the other existing treatments in terms of effect and duration. However, there is no research regarding the effect of ST on cancer-related pain in a pediatric patient. Therefore, validation is needed. Also, more research is needed for assessing the factors that influence the response to treatment.

## References

[1] Bennett MI Effectiveness of antiepileptic or antidepressant drugs when added to opioids for cancer pain: systematic review. Palliat Med 2011;25:553–9. 20671006 10.1177/0269216310378546

[2] Mercadante S Managing difficult pain conditions in the cancer patient. Curr Pain Headache Rep 2014;18:395. 24407750 10.1007/s11916-013-0395-y

[3] Anand KJ Hickey PR Pain and its effects in the human neonate and fetus. N Engl J Med 1987;317:1321–9. 3317037 10.1056/NEJM198711193172105

[4] Porter J Jick H Addiction rare in patients treated with narcotics. N Engl J Med 1980;302:123. 10.1056/nejm198001103020221 7350425

[5] Kanner RM Foley KM Patterns of narcotic drug use in a cancer pain clinic. Ann N Y Acad Sci 1981;362:161–72. 6114697 10.1111/j.1749-6632.1981.tb12804.x

[6] Majithia N Smith TJ Coyne PJ Scrambler therapy for the management of chronic pain. Support Care Cancer 2016;24:2807–14. 27041741 10.1007/s00520-016-3177-3 PMC4973603

[7] Marineo G Untreatable pain resulting from abdominal cancer: new hope from biophysics? JOP 2003;4:1–0. 12555009

[8] Serafini G Marineo G Sabato AF “Scrambler therapy”: a new option in neuropathic pain treatment? Pain Clinic 2000;12:287–98.

[9] Smith TJ Coyne PJ Parker GL Pilot trial of a patient-specific cutaneous electrostimulation device (MC5-A Calmare (R)) for chemotherapy-induced peripheral neuropathy. J Pain Symptom Manage 2010;40:883–91. 20813492 10.1016/j.jpainsymman.2010.03.022 PMC4383258

[10] Ricci M Pirotti S Scarpi E Managing chronic pain: results from an open-label study using MC5-A Calmare (R) device. Support Care Cancer 2012;20:405–12. 21394458 10.1007/s00520-011-1128-6

[11] Ko YK Lee HY Lee WY Clinical experiences on the effect of scrambler therapy for patients with postherpetic neuralgia. Korean J Pain 2013;26:98–101. 23342218 10.3344/kjp.2013.26.1.98 PMC3546221

[12] Attal N Cruccu G Haanpaa M EFNS guidelines on pharmacological treatment of neuropathic pain. Eur J Neurol 2006;13:1153–69. 17038030 10.1111/j.1468-1331.2006.01511.x

[13] Falk S Dickenson AH Pain and nociception: mechanisms of cancer-induced bone pain. J Clin Oncol 2014;32:1647–54. 24799469 10.1200/JCO.2013.51.7219

[14] Pachman DR Weisbrod BL Seisler DK Pilot evaluation of Scrambler therapy for the treatment of chemotherapy-induced peripheral neuropathy. Support Care Cancer 2015;23:943–51. 25245776 10.1007/s00520-014-2424-8 PMC4383262

[15] Park HS Sin WK Kim HY Scrambler therapy for patients with cancer pain—case series. Korean J Pain 2013;26:65–71. 23342211 10.3344/kjp.2013.26.1.65 PMC3546214

[16] Marineo G Iorno V Gandini C Scrambler therapy may relieve chronic neuropathic pain more effectively than guideline-based drug management: results of a pilot, randomized, controlled trial. J Pain Symptom Manage 2012;43:87–95. 21763099 10.1016/j.jpainsymman.2011.03.015

[17] de Leon-Casasola OA Spinal cord and peripheral nerve stimulation techniques for neuropathic pain. J Pain Symptom Manage 2009;38 (suppl):S28–38. 19671469 10.1016/j.jpainsymman.2009.05.005

[18] Foletti A Durrer A Buchser E Neurostimulation technology for the treatment of chronic pain: a focus on spinal cord stimulation. Expert Rev Med Devices 2007;4:201–14. 17359225 10.1586/17434440.4.2.201

[19] Niv D Maltsman-Tseikhin A Lang E Postherpetic neuralgia: what do we know and where are we heading? Pain Physician 2004;7:239–47. 16868598

[20] Polaner DM Taenzer AH Walker BJ Pediatric Regional Anesthesia Network (PRAN): a multi-institutional study of the use and incidence of complications of pediatric regional anesthesia. Anesth Analg 2012;115:1353–64. 22696610 10.1213/ANE.0b013e31825d9f4b

[21] Widmer B Lustig S Scholes CJ Incidence and severity of complications due to femoral nerve blocks performed for knee surgery. Knee 2013;20:181–5. 23276419 10.1016/j.knee.2012.11.002

[22] Morau D Ahern S Management of local anesthetic toxicity. Int Anesthesiol Clin 2010;48:117–40. 20881531 10.1097/AIA.0b013e3181faa464

[23] Salinas FV Joseph RS Peripheral nerve blocks for ambulatory surgery. Anesthesiol Clin 2014;32:341–55. 24882122 10.1016/j.anclin.2014.02.005

